# Experiences and perceptions of perinatal depression among new immigrant Chinese parents: a qualitative study

**DOI:** 10.1186/s12913-021-06752-2

**Published:** 2021-07-26

**Authors:** Qiao Li, Wenqing Xue, Wenjie Gong, Xin Quan, Quanlei Li, Lina Xiao, Dong (Roman) Xu, Eric D. Caine, Ellen L. Poleshuck

**Affiliations:** 1 HER Team and Department of Maternal and Child Health, Xiangya School of Public Health, Central South University, 110 Xiangya Road, Changsha, 410078 Hunan China; 2 Department of Psychiatry, University of Rochester, Rochester, NY 14642 USA; 3 Institute and of Applied Health Research, University of Birmingham, Birmingham, B15 2TT UK; 4 Department of Family Therapy, Nova Southeastern University, Fort Lauderdale, USA; 5 School of Nursing, Johns Hopkins University, Baltimore, USA; 6 East China Institute of Social Development, East China University of Science and Technology, Shanghai, China; 7 ACACIA Lab for Health Systems Strengthening, Institute for Global Health and School of Health Management, Southern Medical University, Guangzhou, China; 8 Departments of Psychiatry and Obstetrics and Gynecology, University of Rochester Medical Center, Rochester, NY 14642 USA

**Keywords:** Chinese immigrants, Perinatal depression, Help-seeking, Qualitative research

## Abstract

**Background:**

Immigrant status, acculturation level, race and ethnicity have been found to contribute to the utilization of mental health services in the perinatal period. This study explored perinatal experiences and perceptions among Chinese immigrant mothers and their spouses, as well as the possible barriers and facilitators that affect their health care utilization.

**Methods:**

We recruited 13 women ages 18–35 years born in mainland China, living in Rochester, New York, and residing less than 5 years in the United States. Participants primary language was Mandarin Chinese and all had given birth to at least one live infant within the past 7 years. Participants’ age was at least 18 years old at the time of delivery. Five spouses also participated. We divided women in two focus groups and held one focus group for men, with data collection including demographic questionnaires and semi-structured focus group questions conducted in December 2014. Data were analyzed following thematic analysis.

**Results:**

Four themes emerged: experiences of perinatal depression; perceptions of perinatal depression; general preventive and coping strategies; and attitudes toward the supportive use social media applications (apps) and text messaging during the perinatal period. Participants had limited knowledge of perinatal depression and had difficulty distinguishing between normal perinatal mood fluctuations and more severe symptoms of depression. They discussed immigrant-related stress, conflicts with parents/in-laws while “doing the month”, the perceived gap between the ideal of “perfect moms” and reality, and challenges with parenting as the causes of perinatal depression. Women approved of screening for the condition but were conservative about follow-up interventions. As for the management of perinatal depression, participants preferred to deal with the problem within the family before seeking external help, due to potential stigma as well as Chinese traditional culture. They were receptive to obtaining pertinent health information from anonymous social media apps, preferring these to personal text messages.

**Conclusion:**

The recent immigrant Chinese parents to the United States in the study had limited knowledge of perinatal depression and did not make full use of mental health services for support due to language and cultural barriers. Screening for perinatal depression is only the first step. Future research should explore what interventions may serve as an acceptable approach to overcoming these gaps.

**Supplementary Information:**

The online version contains supplementary material available at 10.1186/s12913-021-06752-2.

Perinatal depression, defined as depression that occurs during pregnancy or within 12 months after delivery, affects approximately 6.5 to 12.9% of pregnant and postpartum women worldwide [1]. As one of the most common complications of pregnancy and the postpartum period [2], PND jeopardizes maternal health [3, 4] and the growth and development of the offspring [5, 6].

A systematic review and meta-analysis found that immigrant women were twice more likely to experience postpartum depressive symptoms than non-immigrant women [7]. According to a cohort study, for most foreign-born immigrant Chinese mothers, their prevalence of any depressive symptom based on Center for Epidemiological Study-Depression was 35.9% [8]. Isolation and lack of social support and loss of familiar rituals and traditions in the new environment may influence immigrants’ mental health and induce depression [9, 10]. As well, immigrant status, limited acculturation [11], lack of access to resources [12], stigma [13], and language barriers [14] may prevent immigrant women from optimal utilization of medical and mental health services during the perinatal period. Therefore, in order to explore how best to provide culturally attuned, accessible services and improve their mental health it is necessary to explore the experiences of immigrant populations during the perinatal period, as well as their attitudes and preferences for seeking care for mental health .

Individuals with a Chinese background comprise the largest Asian-American group in the United States (US) [15]. We explored in this study how foreign-born Chinese immigrant women in the US, and several of their spouses, dealt with pregnancy and postpartum care, with specific attention to perinatal depression. There are many deeply held Chinese traditions that support new mothers and families which may influence postpartum well-being and mental health [16, 17]. Many Chinese immigrant families seek to retain perinatal Chinese customs such as “doing the month” [18]. “Doing the month” is a time when a new mother is advised to restrict her activities following delivery, including staying at home and taking nutritious food [16, 19]. Typically, mothers and mothers-in-law stay with the family during the month, providing support and advice.

Thus, the purpose of this study was to learn more about Chinese immigrant mothers’ experiences during the perinatal period and to explore possible barriers and facilitators that can affect their use of mental health services.

## Method

We recruited a convenience sample of participants from Rochester, NY, USA. The inclusion criteria were: Chinese women born and raised in mainland China who had resided in the US less than 5 years; ages 18–35 years; able to read and speak Mandarin Chinese as their primary language; having given birth to one or more live infants within the past 7 years in the US or in China; and over 18 years of age when the child was delivered. We excluded anyone unable to engage in the informed consent process. The spouses of the female participants were invited to participate if they were > 18 years, residing with the enrolled mother, able to read and speak Mandarin Chinese as their primary language; the biological father of at least one of the children. We recruited our convenience sample of women by posting recruitment messages on popular Chinese social media applications (apps) (e.g., Weibo, WeChat) used by local Chinese scholars and students in Rochester; posting recruitment flyers at local Chinese schools and churches; promoting referrals from friends and neighbors; and encouraging word of mouth. When potential participants initially contacted us, we reviewed inclusion criteria and invited those who qualified and were interested to attend a focus group. The final sample resulted in 18 participants—13 women and five men. Each participant received a gift-card in recognition of the time devoted to participation. The study was reviewed and approved by the University of Rochester Research Subject Review Board.

We used questionaries to collect demographic information (See Appendix 1 and Appendix 2). Semi-structured focus group guides were developed and conducted by the lead investigator in Chinese Mandarin (WJG), a Chinese obstetrician-gynecologist with training in qualitative research. Another investigator (XQ) assisted conducting the interviews. Interview questions included: knowledge about perinatal depression; recounting personal experiences during pregnancy and the postpartum period; coping with perinatal depressive symptoms, if they had experienced them; defining potential barriers and facilitators that might affect their use of health services in the US; and potentially accepting mobile health applications (apps) as interventions for perinatal depression. (See Appendix 3 and Appendix 4).

Focus groups were conducted in a private, comfortable room conveniently located for the participants, with childcare provided. Women participants were divided into groups of six and seven (FG1 and FG2, respectively) based on convenience of scheduling; the men met once (FG3). Before the formal start of each focus group, interviewees gave verbal informed consent and completed a demographic questionnaire. Each group interview lasted for 60–90 min and was audio-recorded. Two researchers transcribed the interviews verbatim from the audio recordings into Chinese within 4 weeks and kept records of non-verbal information.

Demographic data were summarized by descriptive statistics. The qualitative data were collated and analyzed using MAXQDA 2020 based on thematic analysis [20]: 1) familiarizing with the data, by reading through all the participant accounts several times; 2) generating initial codes to identify important sections of text and attach labels to index them as they relate to a theme or issue in the data; 3) searching for themes by bringing together components or fragments of ideas or experiences; 4) reviewing themes—assessing the coded data extracts for each putative theme to consider whether they appear to form a coherent pattern; 5) defining and naming themes: determining what aspect of the data each theme most clearly captured and identifying what is of interest and why; 6) describing the fundamental structure of the phenomenon; and 7) producing the report.

In this study, the researchers (QL and WQX) first analyzed the interview records in Chinese. To achieve better understanding of participants’ subjective expressions and experiences, the researchers collaborated closely comparing their analyses in a stepwise fashion to achieve consistency and agreement. A team member (XQ) fluent in both Chinese and English translated the analysis results, including codes and quotations, into English. In the last step, a Chinese-English, bilingual researcher (KKC) checked the accuracy of the translation.

## Results

The average age of our 13 female participants was 31.0 ± 4.0 years old and the average length of residence in the United States was 27.1 ± 16.6 months. All women participants were married, had health insurance, and their educational attainment was a bachelor’s degree or higher. The age of the five spouse participants was 31.0 ± 6.0 years old and their average length of residence in U.S. was 28.6 ± 18.7 months, and each had at minimum a bachelor’s degree.

Eleven of the 13 women had nuclear families only in the US; 10 had a household annual income less than $60,000. Their primary daily supports during pregnancy involved their husbands (*n* = 5), themselves (*n* = 4), their mothers (*n* = 3), and their mother-in law (*n* = 1). In contrast, primary postpartum support included their mothers (*n* = 7), mothers-in law (*n* = 5), and husband (*n* = 1). (See Table 1).

**Table 1:** SAMPLE CHARACTERISTICS (*N* = 18)

Characteristics	N	
	WOMEN (***N*** = 13)	MEN (***N*** = 5)
**Age (in years)**		
Mean (SD)	31 ± 4	31 ± 6
Range	25–38	25–40
**Marital Status**		
Married	13	5
**Employment status in the perinatal period**		
Employed	5	2
Unemployed	8	3
**Education level**		
4-year college degree	7	0
Master’s degree	2	1
Doctoral level degree	2	2
Current graduate student	2	2
**Months lived in the US**		
Mean (SD)	27.1 ± 16.6	28.6 ± 18.7
Range	1–50	13–60
**Main caregiver throughout pregnancy for the woman**		
Her mother	3	NA
Mother-in-law	1	NA
Husband	5	NA
Herself	4	NA
**Main caregiver for the woman during the postpartum**		
Her mother	7	NA
Mother-in-law	5	NA
Husband	1	NA
Herself	0	NA
**Whether adhere to the tradition of “Doing the month “**		
Yes, for all my children	3	NA
Yes, for my children born in China only	1	NA
No	7	NA
Other	1	NA
**Whether adhere to the tradition of “Doing the month “**		
Yes	6	NA
No	7	NA
**Way paid for the delivery services**		
Entirely on her own	13	NA
Partly by medical insurance	0	NA
**Family annual income during the most recent pregnancy**		
< $60,000	10	4
≧$60,000	3	1
**Whether have children born in China**		
Yes	3	4
No	10	1
**Whether have children born in the US**		
Yes	10	1
No	3	4
**Family structure**		
Nuclear family	9	4
Dyadic nuclear family	1	0
Live with own parents	2	0
Live with parents-in-law	0	1
Live with child only	1	0

*NA* used to mean “not applicable”

Note: To aid the readers’ comprehension of the results, all quotes presented below, shown in italics, identify the speaker’s participant identification number for each focus group, followed by the focus group (FG) number. Themes were defined as arising in multiple groups, in addition to being expressed by multiple members of the same group only.

### Experiences of perinatal depression

Emotional distress was a theme that emerged in both mother focus groups. Whether the pregnancy was planned or not, the women interviewed generally were happy to learn that they were pregnant: *It wasn’t in the cards, but I was especially happy when I found out I was pregnant.* (04,FG1) However, with the onset of pregnancy, the physical discomfort made it difficult and even depressing for some women: *Serious pregnancy symptoms made me sick, even gave me that depressed feeling. I would vomit when I smelled anything or just drank water*. (01,FG1) Most of the women interviewed reported frequent feelings of stress, fear, and anxiety during the perinatal period due to worrying about the baby: *I suffered from sinusitis ten days after delivery. The doctor advised me to do a CT scan, but I thought CT had radiation and I was really worried that it would influence the breast feeding so I insisted on an MRI at that time. I didn’t feed my baby breast milk when I used antibiotics until the half-life of the antibiotic was gone. The radiation, the antibiotic, everything led to my anxiety.* (02,FG2). Some women become emotionally sensitive during the perinatal period, especially concerned about others’ appraisal of them, contributing to unhappiness, irritability, unprovoked tears or anger: *During pregnancy, I think I became more sensitive and sometimes when someone said something, I would feel like it was directed at me and it upset me. And I would want to cry.*(05,FG1) *Well, I just didn’t feel well at all. My whole state was not very good. I guess I was just very irritable, and it was irritable. I felt down when my baby kept crying. But it was really not because of it. I just felt down for no reason.* (07,FG2) Several expressed the view that women with baseline depression could suffer greater depression during pregnancy. Two women with a history of depression before pregnancy had suspended their antidepressants during pregnancy because of concerns about their adverse effects on the fetus: *I stopped taking them (antidepressants) right after I got pregnant … then I felt like my depressive symptoms worsened*. (04,FG2).

### Perceptions of perinatal depression

#### Partial understanding of perinatal depression

Participants of all three focus groups were familiar with the terms “postpartum depression” or “perinatal depression.” Nearly all identified some depressive symptoms: *Sometimes I was unable to control my emotions and suddenly cried for no reason. And it was hard for me to fall asleep.* (04,FG1).

Four out of five male respondents mentioned they had noticed their partners’ reluctance to communicate as an important sign of depression: *I think the salient feature of the depression is that she didn’t want to talk to you.* (02,FG3) However, they had difficulty distinguishing between normally expressed uncertainties and emotions arising during perinatal period and symptoms indicative of significant perinatal depression: *I could distinguish the differences when it (my wife’s mood) had big changes, but I was not sure whether it could be regarded as depression or not.* (03,FG3).

#### Describing factors associated with perinatal depression

The respondents identified several potentially important factors that could contribute to the development of significant depression based on both their own experiences and what they had observed with others. They described depressed feelings related to being an immigrant, such as having a small social circle, a sense of isolation, and difficulty adjusting to living far from home: *I have heard from my wife that one of her friends lived in an apartment, and there were few friends to communicate with in the perinatal period. She said she felt depressed at that time*. (03,FG3) They also reported that conflict with mothers-in-law can be associated with depression: *I don’t want to recall the first weeks after delivery. My mother-in-law refused to take care of me and my baby. She didn’t help during nighttime as she said the baby didn’t seem happy to be cared for by her at night. So, I had frequent conflict with her during that time*. (01,FG2). Women participants additionally described depressive symptoms associated with conflicts with their elders, especially involving “doing the month “: *If I thought ‘doing the month’ was a good idea, and others suggested that I should do so, that’s fine. However, if I didn’t like the idea, and my mother-in-law kept saying I should, I would feel depressed*. (07,FG2) The men uniformly expressed the same view: *I support my wife, and she can do everything she wants. She can follow what the elders say if she wants it (doing the month), or ignore it if she thinks it’s wrong. If she remains happy, she would recover more quickly If she is not happy, then even taking ginseng wouldn’t help. If I don’t support her, she would be more depressed,* (02, FG3) Lastly, the gap between ideal expectations of parenthood and the realities of caring for a newborn was identified as related to depression. Some of the women interviewed were excited to become a mother before pregnancy, but after the birth they found themselves struggling to cope with the reality: *When I saw pictures of other moms breastfeeding their babies, I thought ‘what a happy scene’. But with the baby in my own arms, screaming hard as if I was abusing him, then all the happy scenes you imagined in the past seemed to just turn into big struggles.* (03, FG1). And: *I thought the baby would be born knowing how to suckle but he didn’t do so until 2 weeks after delivery. I felt so bad about it that I just fell apart.* (04, FG1).

#### Screening for perinatal depression

All women participants approved of universal screening for perinatal depression by their obstetrical providers. They reported depression screening is more acceptable when partnered with other routine prenatal testing, such as general bloodwork, glucose monitoring, or ultrasound exams: *Yeah, they gave me a questionnaire to fill out in at the hospital. I didn’t pay much attention as there were plenty of exams and blood tests anyway during the prenatal visits. It’s just another task in the whole thing.* (03,FG1).

### General preventive and coping strategies identified for perinatal depression

#### Address within the privacy of the family first

Due to a lack of understanding or distrust of mental health professionals, as well as the stigma associated with the traditional Chinese concept of minimizing mental illness, respondents tended to address any mental health concerns first within their family: *People always think that seeing a doctor is a very sensitive matter, especially when you go to see a doctor for depression. You really don’t want others to think that you have problems.* (02,FG3) Participants reported trying to identify strategies to improve their emotional state, such as learning about coping skills or posting their thoughts on virtual forums such as WeChat: *I had some (depressive) symptoms. But the book I read suggested that it had something to do with hormonal changes and it would fade away slowly. So, I just tried to think in a positive way.* (04,FG1). Women identified distraction as a common strategy: *I would choose to work when I am pregnant again because I think I need some distraction. Focusing entirely on the baby may make the pregnancy seem longer and I’d feel worse. Therefore, I preferred working and I was pretty happy working*. (02,FG1) Social support also was identified as protective. Husbands, especially, can help alleviate their wives’ distressed emotions, by providing emotional support or by sharing housework and child care: *It’s comforting that my husband was good at talking. He would talk to me when I was not happy. The role of husband was very important. He talked a lot to me and made me feel that some conflicts in the family can be solved through communication. He was nice to me and I felt peaceful in the perinatal period* (01,FG2) Similarly, sharing feelings with close or trusted family members, such as the new mother’s parents, was valued by both women and men. *To solve this problem (depression), I will find her parents to talk to her if they were around. I think she would like to talk to people she can trust.* (02,FG3).

#### Seek outside help later

When efforts within the family fail to improve perinatal depression, respondents would consider seeking outside help through new social connections or professionals. Participants were encouraged to attend tea parties for mothers in the community and vent their emotions to new friends in US. Through communicating with peers, they learned about parenting and obtained support and comfort from other mothers who also experienced mood fluctuations during the perinatal period: *If it was a friend of mine who was experiencing perinatal mood swings, I’d share my own experience with her, and I thought it might be helpful. Because I had a similar experience, I was more likely to understand what she was feeling.* (04,FG1) For those who still find themselves struggling after talking with friends or family, the women might seek help from a mental health professional if their mood remained distraught or uncontrollable: *I feel like the depression ... If it gets so bad that it becomes unmanageable, I’d probably go to a doctor.* (04,FG1) Participants noted that if a deeply distressed woman continued to decline to see a psychiatrist, her family could try having a psychologist or psychiatrist talk with her outside the clinic: *if it comes to the point of looking for a doctor, my idea may be to say for example, well, getting a psychologist or psychiatrist to meet with my wife and me in places like a café … mainly because I’m afraid she might feel ashamed of her illness in the setting of clinics*. (02,FG3). Respondents generally were not interested in medication as a treatment option: *Maybe the drug is not the best way to cure her, maybe she (my wife) should change the whole way of life.* (03,FG3).

### Attitude towards using text messaging and social media during the perinatal period for health information

#### Receptivity to the use of text messaging and social media as an educational resource

Respondents generally reported that they would be trusting and receptive to educational information published on social media outlets by authoritative organizations or individuals: *I followed what she (a gynecologist from Beijing) said in Weibo (a social media platform), because she is very famous in China. I can learn how to take care of babies there.* (04,FG2) Nearly all of the respondents have used perinatal social media platforms or obtained perinatal health-related information through social media. They expressed a positive attitude toward using these resources during the perinatal period: *It’s good to spread mental health information through such social media apps.* (03,FG3) The social media platforms they accessed cover a wide range of topics, providing pregnant women and their spouses with information including: pregnancy care, estimated due date, post-natal rehabilitation, mental health, and infant feeding. They were described as helping inexperienced mothers and fathers navigate these novel tasks more confidently. *My wife was confused about many things in the perinatal period, and she could get a great deal of useful information from these apps.* (04,FG3) However, when the women and their spouses were experienced with newborns and related postpartum challenges, their use of apps lessened: *The app would be useful with your first child. But after one or 2 weeks, you gradually work out what to do and when to do them. At that time, you would not rely on the app to take care of the baby anymore.* (02,FG3) Language was an obstacle for some when using local resources—whether apps or talking to her doctor: *My wife does not speak very good English. She may have trouble telling her doctor about the changes in her mood during the perinatal period. So, it’d be hard for her doctor to understand her condition*. (03,FG3)” Thus some respondents followed the accounts of specific Chinese doctors through social media apps to obtain needed the information or to ask questions in Chinese: *I followed the Weibo account “I work in hospital” which often publishes some useful information such as, “What should I do if the baby is choking?*” (04,FG2) Additionally, many women joined the mother’s communication groups on WeChat to gain useful parenting tips or encouragement from other women.

#### Expect personalized service

Respondents described a desire to have access to a range of options to learn about perinatal depression: *Every person has her own preference. When she needs the information, you need to give them choices of how such information is conveyed* … *(otherwise) the health messages you send out would be disposed of as junk text messages or junk Email.* (02,FG3) We found that women respondents generally reported they preferred social media apps over text messaging because the former gave them more sources of information to follow, who to follow, when to view it, and greater anonymity: *Text messages make me feel I am a passive recipient. If it’s something on Weibo or WeChat (social media platforms), I can decide whether to read it based on my need. If the information on perinatal health comes from these authoritative sources, I will selectively read it.* (04,FG2) The male respondents indicated that health education information about perinatal depression should be sent directly to mothers, interspersed with infant feeding information: *I think husbands are usually careless, they wouldn’t read (the mental health messages). So, it is better to send the messages directly to the cell phones of the moms. It’d be best giving some parenting tips at the same time. Actually, moms care a lot about their babies’ health conditions.* (04,FG3).

## Discussion

Although perinatal depression was rarely discussed in China in 2014 at that time of this study, several organizations in the U. S were recommending routine screening [21–23]. Although all of our participants had lived most of their lives in China, they all expressed willingness to be screened for perinatal depression. This did not suggest, however, that they were willing to be evaluated further or referred for treatment. They reported they often did not complete questionnaires nor want referrals for service. A systematic review by our team has identified similar issues in many countries [24] and there is a serious lack of data on perinatal depression screening and referral patterns in China or among Chinese members of other societies. Next steps needed include determining culturally attuned ways to deliver perinatal depression care to Chinese immigrants.

The qualitative findings in this study offer potential directions for future research. Consistent with what has been reported previously [25–28] our findings reflect attitudes in Chinese culture regarding the shame associated with mental health concerns and women’s reticence to reveal their distress. It also underscores the priority placed on a private and family-first approach to resolving problems if they are, in fact, shared outside of the family at all [29, 30]. Perinatal depression screening now is being advocated in China [31], but there remains insufficient data regarding how best to accomplish effective identification and subsequent referrals to care for those women who need it the most. The well-known stigma associated with help-seeking is compounded by the relative lack of training among Chinese health providers in dealing with perinatal depression [32], a deficiency that now is being more actively addressed in the U.S. and other western nations.

Chinese immigrants such as our participants express doubts about the role and value of mental health professionals (e.g., psychologists, psychiatrists and others), describing evident cultural and educational barriers in fundamental terms: *The professionals cannot really improve my situation*. Language limitations add further to the cultural barriers confronting immigrant women when seeking health services [14]; they are reluctant to speak about their emotional problems and worry their doctors will not understand their symptoms or concerns. The tendency of women of Asian cultural backgrounds to less frequently seek help for perinatal depression than other immigrant women [33] may be the result of a combination of these factors.

A strength of our study derives from the involvement of a few fathers. Most of our women participants, as well as those in another study [34], were hopeful that perinatal depressive symptoms would subside naturally over time. The Chinese immigrant men, on the other hand, tended to take initiative and believed that they, as spouses, played an important role in detecting their wives’ mood disorders and providing support for them. These Chinese husbands provided social support to their wives by sharing household chores, taking care of children, and reconciling conflicts between their mothers and their wives. The husbands thought health education about perinatal depression was desirable and that knowledge of mental health should be integrated with parenting tips, consistent with women’s view in another study [35]. The husbands suggested sending health education information only to their wives because they were tended to ignore this information. Unlike their wives, who were more concerned about the development of depression, husbands were more concerned with how deal with it once it was present. While their perspective was supportive and action oriented, it was less attuned to initial exacerbating circumstances and thus less likely to preventing the initial emergence or recurrence of depression.

It remains uncertain whether ‘doing the month’ is a risk factor for perinatal depression or serves as a protective factor in some fashion [36, 37]. Although the women in our study generally reported “doing the month”, their implementation differed from how it is traditionally observed. For example, they took a shower and washed their hair during the month, which were not allowed traditionally. Our participants did not reject the idea of ‘doing the month,’ despite being educated and having lived in the U.S. for a longer period of time than reported by Ta Park VM [18], but they reported considerable stress related to implementing it in the US. They acknowledged a wish to be cared for by their family, especially their mother or mothers-in-law. They encountered difficulties when being advised to “do the month” according to the traditions of elder women rather than following their own ideas. These conflicts between the respondents and their elders served as the major reported source of emotional fluctuations.

The high level of acceptance of using social media to obtain health information among these Chinese immigrant women and their spouses is similar to other studies [30, 38]. Even in 2014, use of social media was common practice throughout China, a nation with a many decades’ of internal migration and the consequent need for inexpensive long-distance communication. Our immigrant participants were very familiar with social media tools and relied heavily on social media to keep in touch with friends and family in China. Apps could serve as a platform for telehealth, as they offer a level of anonymity that may reduce the stigma women feel when face-to-face contact is not required, as noted previously [39]. They can help overcome language barriers, allowing couples to seek help from specialists in China or from distant centers internationally. Our participants used social media apps for parenting and physical health, but rarely for mental health concerns, similar to previously reported results [40, 41]. They also were receptive to a perinatal depression intervention using social media apps. We caution, however, that there may be a significant gulf between expressed acceptance and actual use.

We see several shortcomings in our study. Ours was a self-identified convenience sample, which may lead to selection bias. All participants held bachelor’s degrees or higher, and we have no way to infer extrapolation to less educated women and men. Inherently the study potentially was subject to recall bias; nine of the 13 women were not in the perinatal period when they participated in the focus group interviews. We set a seven-year limit on the time since birthing their last baby, but we cannot be assured that this interval was sufficiently short to avoid bias. We did not have access to their medical records and had no way of confirming any results from their perinatal depression screening, leaving us to depend solely on their recall of what they had experienced and the extent of their reported distress. Lastly, during the interviews we used the term “mood” in eliciting participants’ experiences with perinatal depression which may have been too narrow. Incorporating terms like “stress” and “worry” to may have elicited a broader range of experiences. We also focused on soliciting feedback about the use of social media as a resource rather than asking more broadly about what types of support respondents believed would be most useful.

Taken together, our results point out the need for clinicians in the US who work with Chinese women to recognize that screening for perinatal depression is only the first step. They must be proactive and thoughtfully inquire about emotional distress and interpersonal conflict, while respecting cultural sensitivities about disclosing symptoms of mental disorders. The culture of strong family focused values, especially pertaining to husbands, mothers, and mothers-in-law, suggests clinicians must carefully navigate and balance women’s need for privacy and tradition-based expectations of family openness and family-based decision making. As mentioned above, Chinese immigrant women report acceptance of depression screening, but are reluctant to accept referrals for care. This suggests that online resources for women at high risk of perinatal depression may be more accessible and acceptable. Finally, our results are qualitative and very preliminary, which in turn emphasizes the need for more research regarding perinatal depression among women of diverse Asian backgrounds, and the additional burdens associated with giving birth in a nation and culture far from their original home.

## Supplementary Information

**Additional file 1.**

**Additional file 2.**

**Additional file 3.**

**Additional file 4.**

## Data Availability

The data generated and/or analyzed during the current study are not publicly available due to restrictions related to confidentiality i.e., they contain information that could compromise the privacy of research participants, but are available from the corresponding author WJG on reasonable request.

## References

[1] Gavin NI, Gaynes BN, Lohr KN, Meltzer-Brody S, Gartlehner G, Swinson T (2005). Perinatal depression: a systematic review of prevalence and incidence. Obstet Gynecol.

[2] Committee Opinion No ACOG (2018). 757: screening for perinatal depression. Obstet Gynecol.

[3] Sit D, Luther J, Buysse D (2015). Suicidal ideation in depressed postpartum women: Associations with childhood trauma, sleep disturbance and anxiety. J Psychiatr Res.

[4] Kendig S, Keats JP, Hoffman MC, Kay LB, Miller ES, Moore Simas TA, Frieder A, Hackley B, Indman P, Raines C, Semenuk K, Wisner KL, Lemieux LA (2017). Consensus bundle on maternal mental health: perinatal depression and anxiety. Obstet Gynecol.

[5] Netsi E, Pearson RM, Murray L, Cooper P, Craske MG, Stein A (2018). Association of persistent and severe postnatal depression with child outcomes. JAMA psychiatry.

[6] Kingston D, McDonald S, Austin MP, Tough S (2015). Association between prenatal and postnatal psychological distress and toddler cognitive development: a systematic review. PLoS One.

[7] Falah-Hassani K, Shiri R, Vigod S, Dennis CL (2015). Prevalence of postpartum depression among immigrant women: a systematic review and meta-analysis. J Psychiatr Res.

[8] Huang ZJ, Wong FY, Ronzio CR, Yu SM (2007). Depressive symptomatology and mental health help-seeking patterns of U.S.- and foreign-born mothers. Matern Child Health J.

[9] Dankner R, Goldberg RP, Fisch RZ, Crum RM (2000). Cultural elements of postpartum depression. A study of 327 Jewish Jerusalem women. J Reprod Med.

[10] Ayers JW, Hofstetter CR, Usita P, Irvin VL, Kang S, Hovell MF (2009). Sorting out the competing effects of acculturation, immigrant stress, and social support on depression: a report on Korean women in California. J Nerv Ment Dis.

[11] Ji P, Duan C (2006). The relationship among acculturation, acculturation stress, and depression for a Korean and a Korean-American sample. [the relationship among acculturation, acculturation stress, and depression for a Korean and a Korean-American sample.]. Asian J Couns.

[12] O'Mahony J, Donnelly T (2010). Immigrant and refugee women's post-partum depression help-seeking experiences and access to care: a review and analysis of the literature. J Psychiatr Ment Health Nurs.

[13] Bilszta J, Ericksen J, Buist A, JJAJoAN M (2010). Women's experience of postnatal depression - beliefs and attitudes as barriers to care.

[14] Sambrook Smith M, Lawrence V, Sadler E, Easter A (2019). Barriers to accessing mental health services for women with perinatal mental illness: systematic review and meta-synthesis of qualitative studies in the UK. BMJ Open.

[15] Bureau USC (2017). Facts for features: Asian-American and Pacific islander heritage month.

[16] Liu YQ, Maloni JA, Petrini MA (2014). Effect of postpartum practices of doing the month on Chinese women's physical and psychological health. Biol Res Nurs.

[17] Wong J, Fisher J (2009). The role of traditional confinement practices in determining postpartum depression in women in Chinese cultures: a systematic review of the English language evidence. J Affect Disord.

[18] Ta Park VM, Goyal D, Suen J, Win N, Tsoh JY (2019). Chinese American Women's experiences with postpartum depressive symptoms and mental health help-seeking behaviors. MCN Am J Matern Child Nurs.

[19] Holroyd E, Katie FK, Chun LS, Ha SW (1997). “doing the month”: an exploration of postpartum practices in Chinese women. Health Care Women Int.

[20] Morrow R, Rodriguez, Alison, King, Nigel (2015). Colaizzi’s descriptive phenomenological method. Psychologist.

[21] U.S. Preventive Services Task Force. Screening for depression in adults: U.S. preventive services task force recommendation statement. Ann Intern Med. 2009;151:784–92. 10.7326/0003-4819-151-11-200912010-00006 19949144

[22] National Association of Pediatric Nurse Practitioners. NAPNAP position statement on the PNP's role in supporting infant and family well-being during the first year of life. J Pediatr Health Care. 2011;25:9a–11a. 10.1016/j.pedhc.2010.10.004 21416738

[23] Earls MF (2010). Incorporating recognition and management of perinatal and postpartum depression into pediatric practice. Pediatrics..

[24] Xue WQ, Cheng KK, Xu D, Jin X, Gong WJ (2020). Uptake of referrals for women with positive perinatal depression screening results and the effectiveness of interventions to increase uptake: a systematic review and meta-analysis. Epidemiol Psychiatr Sci.

[25] Kim SC, Suzuki L, Toyama K, Lei N (2017). Counseling eastern Asian American women. Handbook of counseling women, 2nd ed.

[26] Chen AW, Kazanjian A, Wong H (2009). Why do Chinese Canadians not consult mental health services: health status, language or culture?. Transcultural Psychiatry.

[27] Tabora BL, Flaskerud JH (1997). Mental health beliefs, practices, and knowledge of Chinese American immigrant women. Issues Mental Health Nurs.

[28] Ying YW, Miller LS (1992). Help-seeking behavior and attitude of Chinese Americans regarding psychological problems. Am J Community Psychol.

[29] Abe-Kim J, Takeuchi D, Hwang WC (2002). Predictors of help seeking for emotional distress among Chinese Americans: family matters. J Consult Clin Psychol.

[30] Gong W, Jin X, Cheng KK, Caine ED, Lehman R, Xu DR. Chinese Women's Acceptance and Uptake of Referral after Screening for Perinatal Depression. Int J Environ Res Public Health. 2020;17(22):8686. 10.3390/ijerph17228686. 10.3390/ijerph17228686 PMC7700456 33238480

[31] Commission GOotNH (2020). Notice of the general Office of the National Health Commission on exploring the implementation of special Services for the Prevention and Treatment of depression and senile dementia.

[32] Zhong B-LC J, Chen H-H, Zhang J-F, Xu H-M, Fan Y-P, Wang P-H, Zhou L (2011). Investigation of postpartum depression among medical staff from 21 maternal and child care Ceters in Wuhan: a cross-sectional survey. Chin J Obstet Gynecol Pediatr.

[33] Goyal D, Wang EJ, Shen J, Wong EC, Palaniappan LP (2012). Clinically identified postpartum depression in Asian American mothers. J Obstet Gynecol Neonatal Nurs.

[34] Sword W, Busser D, Ganann R, McMillan T, Swinton M (2008). Women's care-seeking experiences after referral for postpartum depression. Qual Health Res.

[35] Bhat A, Hoeft T, McCoy E, Unutzer J, Reed SD (2019). Parenting and perinatal depression: meeting women's needs. J Psychosom Obstet Gynaecol.

[36] Liu YQ, Petrini M, Maloni JA (2015). "doing the month": postpartum practices in Chinese women. Nurs Health Sci.

[37] Ding G, Niu L, Vinturache A, Zhang J, Lu M, Gao Y, Pan S, Tian Y, Shanghai Birth Cohort Study (2020). "doing the month" and postpartum depression among Chinese women: a Shanghai prospective cohort study. Women Birth.

[38] Wang N, Deng Z, Wen LM, Ding Y, He G (2019). Understanding the Use of Smartphone Apps for Health Information Among Pregnant Chinese Women: Mixed Methods Study. JMIR Mhealth Uhealth.

[39] Watterson JL, Walsh J, Madeka I (2015). Using mHealth to improve usage of antenatal care, postnatal care, and immunization: a systematic review of the literature. Biomed Res Int.

[40] Larsson M (2009). A descriptive study of the use of the internet by women seeking pregnancy-related information. Midwifery..

[41] Hilty DM, Ferrer DC, Parish MB, Johnston B, Callahan EJ, Yellowlees PM (2013). The effectiveness of telemental health: a 2013 review. Telemed J e-health.

