# Supplementary material for: Experiences and perceptions of perinatal depression among new immigrant Chinese parents: a qualitative study
Source: BMC Health Serv Res. 2021 Jul 26;21:739. doi: 10.1186/s12913-021-06752-2 (PMC8311906; doi:10.1186/s12913-021-06752-2)
Supplement: Supplementary file 1 — Additional file 1. [file 12913_2021_6752_MOESM1_ESM.docx]

Appendix 1 Demographic information for mothers

Please answer the following questions. All your answers are anonymous and cannot be connected with you. The information will be used for for this study only.

Please circle the responses that are best for you.

Sex： female male

Age: __________ years

Marital Status:

married

Single

Divorced/separated

widowed

Educational level 1. less than high school 2. High school 3. 4-year College degree 4. Master’s degree 5. doctoral level degree 6. Current graduate student

How long have you lived in the United States? _______ months __________ years

Who lives in your household:

relationship to you age

____________________________________________________________

____________________________________________________________

____________________________________________________________

____________________________________________________________

____________________________________________________________

____________________________________________________________

Do you have children born in China? Y/N If yes, how many? _______

Do you have children born in US? Y/N, If yes, how many? _____________

Who was you main person who supported you throughout your pregnancy？ your own mother, mother-in-law, husband, or other: ________________

Who was you main person who supported you during the postpartum？ your own mother, mother-in-law, husband, or other: ________________

Did you or your partner adhere to the tradition of "yuezi" - self-confinement at home postpartum (do you want them to describe specifically what they did?)

Yes, for all my children (if you have more than one child)

Yes, for my children born in China only

No

Other: ________________________________

Had you ever heard about "postpartum depression" or "Perinatal depression"? Y/N

Did you maintain your job during pregnancy? Y/N

Who paid for the delivery services? - entirely on your own or partly by medical insurance?

Was your family annual income during your most recent pregnancy? more than $60,000 USD/year? Less than $60,000 USD/year?
