# Supplementary material for: Experiences and perceptions of perinatal depression among new immigrant Chinese parents: a qualitative study
Source: BMC Health Serv Res. 2021 Jul 26;21:739. doi: 10.1186/s12913-021-06752-2 (PMC8311906; doi:10.1186/s12913-021-06752-2)
Supplement: Supplementary file 3 — Additional file 3. [file 12913_2021_6752_MOESM3_ESM.docx]

Appendix 3 Abbreviated focus group guide for mothers

1. I’d like for us to get to know each other a little bit. Would you telling me your first name, where in China you are from, how many children you each have, and what ages they are?

2. In your view, how do experiences of pregnancy and delivery compare between China and. in US? (Queries: how are the experiences the same? How are they different?)

3. Tell me how you think pregnancy affects a mother’s mood, if at all? Are there differences in China and in the US as to the way women’s mood may be affected by pregnancy?

4. How do experiences of having a new baby at home compare between China and the US? (Queries: how are the experiences the same? How are they different?)

5. Have you ever known someone who had significant difficulties feeling down or sad, lack of motivation, poor energy, or other similar difficulties during or after pregnancy? What was that like for her?

6. How did others around her respond to the difficulties she was having?

7. Of the women you know who had difficulties, did they ever seek help? If so, what type of help did they seek? And how did that work out for them?

8. If not, what is your understanding of why not? And how did they try to cope?

9. In your opinion, what would be most helpful for someone who is having difficulties with her mood or functioning during or after pregnancy? (Queries: can you give me an example? Who should provide that help?)

10. How do you think “yuezi” practice of postpartum home confinement affects the mood of new mothers? (Queries: what might be helpful about it? What might be problematic about it?)

11. We are trying to find ways to help women learn more about common mood changes during pregnancy and post partum periods. One idea we had was to send electronic messages on cell phones during pregnancy, educating women about mood changes, when it might be a problem and strategies about how you may tackle it? What do you think of the idea? (queries: how do you think women would respond? What might they like and dislike about it? What format might be most helpful: text, phone call, or email)

12. What other suggestions do you all have as to what might be most helpful for Chinese women experiencing mood changes during pregnancy and postpartum periods?
