# Supplementary material for: Experiences and perceptions of perinatal depression among new immigrant Chinese parents: a qualitative study
Source: BMC Health Serv Res. 2021 Jul 26;21:739. doi: 10.1186/s12913-021-06752-2 (PMC8311906; doi:10.1186/s12913-021-06752-2)
Supplement: Supplementary file 4 — Additional file 4. [file 12913_2021_6752_MOESM4_ESM.docx]

Appendix 4 Abbreviated focus group guide for husbands

1. I’d like for us to get to know each other a little bit. Would you telling me your first name, where in China you are from, how many children you each have, and what ages they are?

2. In your view, how do experiences of pregnancy and delivery for a woman compare between China and in US? (Queries: how are the experiences the same? How are they different?)

3. Tell me how you think pregnancy affects a mother’s mood, if at all? What do you know about perinatal mood changes?

4. Are there differences in China and in the US as to the way women’s mood may be affected by pregnancy?

5. In your opinion how the line between the normal mood change and problem in the mood for a woman in her pregnancy or after the delivery can be identified?

6. A case study: A women of 7-month pregnancy exhibits the following things: always staying at home, lack of interests in any activities, feeling down and no energy, lack of attention even when watching TV, sobbing quietly sometimes by the window, worrying about the health of fetus, self-blaming for not being able to enjoy pregnancy like other women, difficult to sleep and waking up early, slow response in conversations. Please help judge whether she has any mood problems, and with what sign did you identify those programs?

7. For this case, what kind of help do you think she should seek? What role should the husband play in this process.

8. What may be the obstacles for her to seek help and how can she overcome it?

9. How do you think “yuezi” practice of postpartum home confinement affects the mood of new mothers? (Queries: what might be helpful about it? What might be problematic about it?)

10. We are trying to find ways to help women learn more about common mood changes during pregnancy and post partum periods. One idea we had is to send text messages on cell phones during pregnancy, educating women’s family members about mood changes, when it might be a problem and strategies about how you may tackle it? How do you like this idea? (questions: If your wife gets pregnant again, will you be willing to receive the text message like this and converse with your wife? What kind of content will you most like to receive? Will the husband be willing to accept this method? What modality of messaging may help you most: text, phone call, or email？Is it better to send to women directly or send to their partners?)

11. What other suggestions do you all have as to what might be most helpful for Chinese women and her families experiencing mood changes during pregnancy and postpartum periods?
